# Cooling Effect of Rivers on Metropolitan Taipei Using Remote Sensing

**DOI:** 10.3390/ijerph110201195

**Published:** 2014-01-23

**Authors:** Yen-Chang Chen, Chih-Hung Tan, Chiang Wei, Zi-Wen Su

**Affiliations:** 1 Department of Civil Engineering, National Taipei University of Technology, Taipei 10608, Taiwan; E-Mail: yenchen@ntut.edu.tw; 2 Information Division, Agricultural Engineering Research Center, Taoyuan 32061, Taiwan; E-Mail: chtan@aerc.org.tw; 3 Experimental Forest, National Taiwan University, No.12 Chien-Shan Rd. Sec.1 Jushan Township, Nantou 55750, Taiwan; 4 Department of Civil Engineering, National Taipei University of Technology, Taipei 10608, Taiwan; E-Mail: chen.yenchang@gmail.com

**Keywords:** flood plain, land surface temperature, remote sensing, river surface temperature, wetland, urban

## Abstract

This study applied remote sensing technology to analyze how rivers in the urban environment affect the surface temperature of their ambient areas. While surface meteorological stations can supply accurate data points in the city, remote sensing can provide such data in a two-dimensional (2-D) manner. The goal of this paper is to apply the remote sensing technique to further our understanding of the relationship between the surface temperature and rivers in urban areas. The 2-D surface temperature data was retrieved from Landsat-7 thermal infrared images, while data collected by Formosat-2 was used to categorize the land uses in the urban area. The land surface temperature distribution is simulated by a sigmoid function with nonlinear regression analysis. Combining the aforementioned data, the range of effect on the surface temperature from rivers can be derived. With the remote sensing data collected for the Taipei Metropolitan area, factors affecting the surface temperature were explored. It indicated that the effect on the developed area was less significant than on the ambient nature zone; moreover, the size of the buffer zone between the river and city, such as the wetlands or flood plain, was found to correlate with the affected distance of the river surface temperature.

## 1. Introduction

With the development and expansion of metropolitan areas, exhaust fumes and heat accumulate. The consequence of this is an urban heat island (UHI) effect. The magnitude and extent can reach up to 12 °C and 8,067 km^2^ [1]. As sustainable urban development becomes an important issue, urban environmental quality is gaining traction in Taiwan. Land use affects the variations in surface temperature, as demonstrated by previous satellite image analysis: Higher temperature in the UHI was located with a scatter pattern which was related to certain land-cover types [2]; the surface temperature was low when the land was used for agriculture. While being developed, the soil was exposed and thus the surface temperature rose. It was found the developing land temperature was 3.8 °C higher than that of the ambient area. After the development was approaching its completion, trees and grass were planted to lower the ambient temperature. At this stage, the developed area was found to be 2.6 °C warmer than its surrounding area. A previous study found the development of agricultural land would raise the surface temperature [3]. Moreover, the growth cycle of crops also affects the surface temperature; for example, rice paddies absorb heat from their adjacent areas and thus result in their cooling. Further, at different stages of crop growth, the cooling effect also varies. Studies showed the cooling effect is strongest at the impounding period, having such an effect at the initial stage of growth. The period of transplanting rice seedlings is the second strongest among all stages [4]. Rice paddy fields in the low-density metropolitan areas of Tokyo have a range of effect of around 150 meters; however, the smaller the paddy area, the less its range of effect is [5]. The intensity of the urban heat island is found to be proportional to the logarithm of population and inversely proportional to the wind speed under cloud-free conditions; that is, as the urban population increases, the decrease in wind speed raises the temperature of the urban environment [6]. The wide use of concrete and asphalt, exacerbated by the solar incidence and artificial heat, causes the temperature in urban areas to be 0.5 ± 1.08 °C higher than suburban areas [7]. 

Due to the diverse factors affecting the urban heat island and their complicated relationships, many approaches have attempted to study these phenomena. The major ones are the meteorological station method, point observation method, moving transect method, and the remote sensing method. The advantage of fixed meteorological stations includes the high data resolution, while the collected data can be used for comparing temperature trends. However, measuring the temperatures of a large urban area requires a significant number of stations. The number of stations is limited due to economic considerations and other associated factors, while most are established outside the range of human activities which might disturb the data collection. Thus, the data from fixed stations can only be used as a supplement for reference or verification while these data alone are not sufficient to be used to conduct analysis [8]. The moving observation method typically uses instruments installed on vehicles. These instruments require proper isolation to prevent interference from ambient thermal sources. These vehicles are driven through cities and suburban areas to collect data [9]. With proper planning, it is an adequate way of data collection for small scale urban temperature studies [10]. However, the urban traffic condition varies; thus, this method is unable to collect data simultaneously for different areas in a city. The remote sensing method adopts satellite images to study surface temperature and compare the difference between urban and suburban areas. This method first converts the radiation intensity data within the infrared window channel into a brightness temperature for the urban and suburban areas, and is then collected by a radiometer mounted on satellites or airplanes. Next, it relates the surface or air temperature data, as measured by surface meteorological stations, with the bright temperature through regression analysis. The advantage of such a method is that it allows simultaneous collection of surface temperature data over large areas at lower cost. The data is representative, while it is also suitable for short-term and long-term analysis. Past studies had been reviewed and demonstrated that the thermal sensors of remote sensing had made some progress and expected to have more progress with the improvements in the spatial and spectral resolutions [11], and less attention paid on the derivation of UHI parameters from land surface temperature and use of remote techniques to estimate urban surface energy modeling [12]. Nevertheless, with the rapid developing technology and progress for Geographic Information System (GIS), three dimensional GIS can manipulate and visualize the spatial data in urban environment which reflect the detailed street reality and provide enough information for the urban climate modeling [13]. Coupled with Multicriteria Decision Making (MCDM) techniques, GIS is enhanced to help analysts to select and implement the suitable MCDM for the problems of handling spatial data [14]. An example for evaluating the spatiotemporal dynamics and urban land-use transformation of Shanghai metropolitan area, with remotely sensing and GIS, showed the potential to monitor and detect the urbanization process [15]. 

Greenbelts and water bodies have the capacity to mitigate temperature variation due to their evapotranspiration [16,17,18]. Moreover, by adopting different strategies, the temperature in the hot and humid metropolitan areas can be effectively lowered [19,20]. Hathaway and Sharples [21] used data collected at fixed meteorological stations to analyze how effectively urban streams lowered the temperature increase from an urban heat island in Sheffield, England. It was found that levees, stream temperature, solar incidence, wind speed, and also relative humidity are the factors affecting temperature variation. The study also found the average reduction can reach about 1.5 °C in the daytime of spring. However, such a cooling effect is reduced as the stream temperature rises. Most studies do not provide direct evidence about how stream water affects the temperature variation in urban areas where the land use condition is very diverse. This study attempts to integrate satellite remote sensing images of multi-sensors for different spatial resolutions to get more details of land use type, together with ground meteorological observation, to analyze the relation between the satellite radiation data and the surface temperature of diverse land use in urban area. Further, using this relationship as a basis and also simulating stream gridding, the range of effects and factors influencing the relation between the rivers and temperatures of urban environment can be determined. This result is verified by applying the findings to a simulation of the condition of Taipei’s metropolitan area.

## 2. Method

### 2.1. Land Surface Temperature and Ground Emissivity Using Landsat 7 Data

The Landsat-7 satellite orbits the Earth at an altitude of 705 km with an inclination of 98.2°. It scans the same area every sixteen days. The Enhanced Thematic Mapper plus (ETM+) on the satellite collects the data of reflected solar incidence from Earth using eight channels of visible and infrared light. Each scene has 5,996 lines and every line has a resolution of 6,320 pixels. The number of high-resolution ground images taken daily is 250. The images can be used for research, as well as constructing a database for sunny and cloudy conditions. It takes 26.31 seconds for Landsat-7 to generate one scene. Each two sequential scenes have 7.3% of overlay and this number increases as the scenes move toward the two poles. For Taiwan, the overlay is 14%. Since 31 May 2003, the scan line correction cannot be executed and thus the images generated since then include a serrated black strip, which has dimensions of one pixel in width and 60 meters in length. Even though this defect can be interpolated by software, the radiation data at the thermal infrared band cannot be made up through such processing. Thus, the damaged area of each image is not used for the calculation of this study; that is, the damaged part is neglected in retrieving surface temperature.

The surface temperature is estimated through the following steps. The reflected radiation from the surface object, measured by Landsat-7/ETM+ at band 6 (6H), is collected as an 8-bit greyscale image with a grade of level 1G. The digital number (DN) is then converted into the magnitude of long-wave radiation, *L_6,TOA_*, at the top of atmosphere by radiation correction:

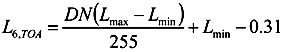
(1)
where *L_max_* is the maximum radiation of the solar spectrum for each scene, while *L_min_* is the minimum radiation of the solar spectrum. For 6H, *L_max_* = 12.65 and *L_min_* = 3.2.

The radiation intensity of spectrum is converted to satellite brightness temperature by Planck’s law:

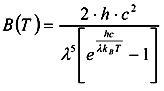
(2)
where *B*(*T*) is spectral radiance; *h* is Plank constant; *c* is speed of light; *λ* is wavelength; *k_B_* is Boltzmann constant; *T* is absolute temperature of the black body. The assumption of Planck’s law is the electromagnetic radiation of an object only relates to its temperature if it is a blackbody. Therefore, the radiation intensity can be converted to the radiative temperature at the top of atmosphere. After inputting the parameters into Equation (2), the formula becomes:

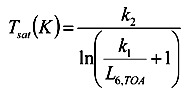
(3)
where *T_sat_* is the radiative temperature at the top of atmosphere, which is also sometimes called the satellite radiant temperature; *k_1_* = 666.09 W/(m^2^-sr-ìm) and *k_2_* = 1,282.71 K.

Based on Stefan Boltzmann law that the total energy radiated per unit surface area of a black body per unit time is directly proportional to the fourth power of the black body’s thermodynamic temperature, the integral for the full spectrum of wavelength can be transferred into the radiance of the long-wave on the top of the atmosphere, 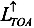:

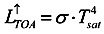
(4)
where σ is the Stefan-Boltzmann constant. 

The upward radiation, received by the satellite sensors, comes from the one emitted by the surrounding medium of atmosphere, 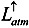, and also the one from the surface of the Earth that passes through the atmosphere. Via atmospheric correction, 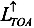 can be expressed by the long wave emissivity of the Earth’s surface, *L^↑^*:

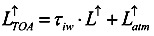
(5)
where *τ_iw_* is the atmosphere transmission coefficient at the thermal infrared band. Since the upward thermal infrared radiation at the surface includes the surface reflection of downward radiation, 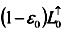, and also the surface radiation, 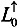 , the equation can be written as:

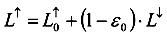
(6)
where *ε_0_* is the spectrum emission coefficient. Thus, it can be found that:

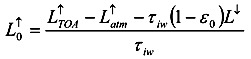
(7)
where *L^↓^* is the downward long-wave radiance [W/m^2^] reaching the Earth surface and *γ_0,iw_* is the surface emissivity at the thermal infrared band. With the consideration of energy balance while viewing the Earth surface as opaque, *i.e.* surface transmissivity *τ_0_* = 0, the surface absorbance *α_0_* equals *ε_0_* and therefore *γ_0,iw_* = (1 - *α_0_* ) = (1 - *ε_0_*).

Viewing the eEarth surface as opaque, then surface reflectivity, *ε*, is considered Equation (8) while the radiance of the long-wave on the top of atmosphere, 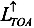 remains as Equation (4). Due to lack of radiosonde data for the area studied, it is simply assumed surface radiation, 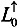, equals the radiation on the top of the atmosphere, 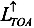. Thus, the land surface temperature, *T_0_*, can be found by Equation (9):

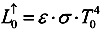
(8)

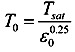
(9)


**Figure 1:**
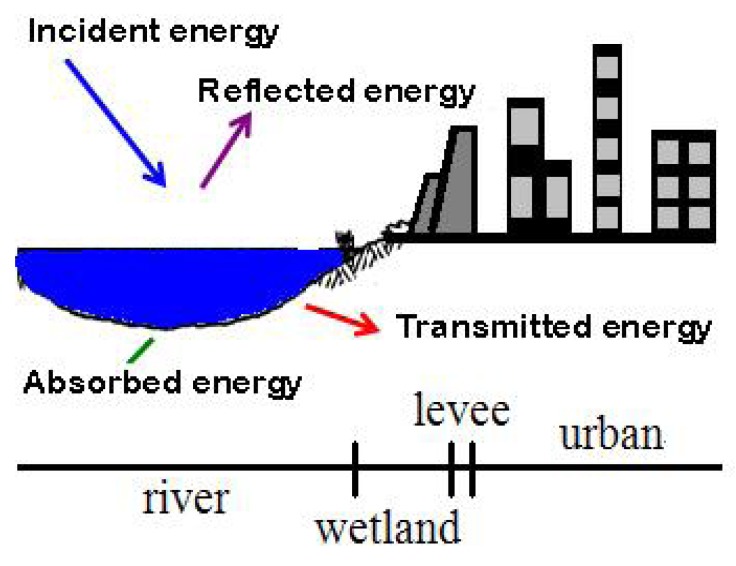
The schematic diagram of solar radiation balance.

The surface temperature is affected by the solar incidence and reflection and can be used to calculate the surface energy flux, as shown in Figure 1. Theoretically, the incidence equals the sum of the reflection, absorption, and transmission. The emissivity is difficult to estimate because the reflectivity changes according to the wavelength of the incident light. For example, such a property for distilled water has a curvilinear relationship between 10 and 12 μm, which causes a small fluctuation on reflectivity. Nevertheless, a real water body contains a significant amount of impurities and thus impairs the estimation of reflectivity. Thus, for each land use category, its reflectivity has to be estimated individually. Further, due to the variation in resolution, each pixel might even contain several types of land use, each having its own reflectivity. The long-wave radiation reflectivities of different land use such as water, bare soil, construction, ponding paddy, wetland, bare paddy, herbal and grass paddy are 0.99, 0.93, 0.93, 0.96, 0.97, 0.92, 0.96 and 0.94, respectively.

### 2.2. Land Use Classification Using Formosat-2 Data

The high resolution of the satellite images eases the interpretation of land use and its categorization. The classification is achieved using the data from Formosat-2, which passes over Taiwan every twelve hours. However, since the visible light band cannot collect image at night time, the useful data can be collected once daily and this information is useful for long term monitoring of surface variation.

**Figure 2:**
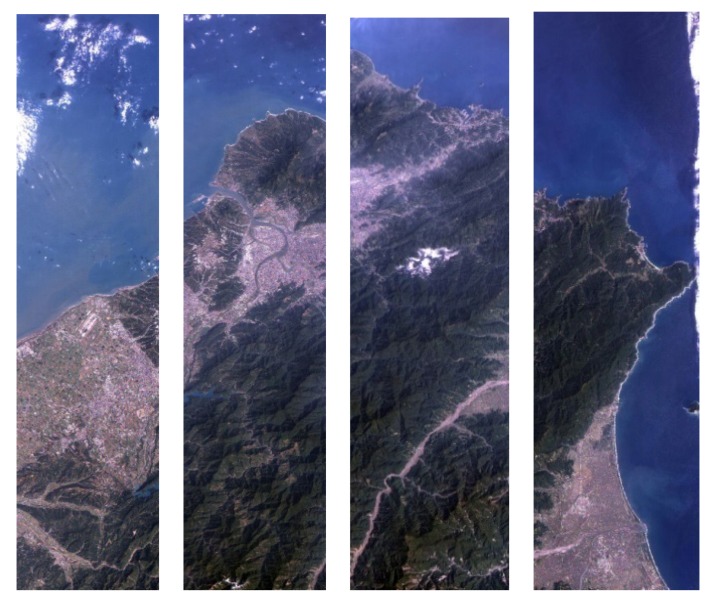
The satellite image bands from Formosat-2.

The image of the study area is acquired by first consolidating four swath images, as shown in Figure 2, followed by categorizing land use for each image. The image classification analyzes and classifies the spectrum digital number (DN) of each pixel on the image by a specific calculation and statistical method. Each Formosat-2 image has to be processed separately as the mosaic images will result in the loss of the original spectrum and cause an inaccurate outcome for the later surface temperature data retrieval.

To increase the accuracy of land classification, the unsupervised classification ISODATA (Interactive Self-Organizing Data Analysis Technique Algorithm) [22] is adopted for this study. The land use of metropolitan Taipei is classified as water, bare land, herbal, construction, and plant. After land classification is performed for the satellite images, 259 points were randomly selected to be compared with high-resolution aerial photos. The result is listed in Table 1, with an overall accuracy of 88.4% and kappa value of 0.85, respectively. It shows good agreement with the ground truth except slight misclassification of bare soil, construction and plant mixed with other classes. Due to the limitation of the pixel solution and the band of the Formosat-2 image, the land classification is yet to yield highly accurate results by high-resolution satellite image. The result of land classification is then used for surface temperature retrieval with the Landsat-7 data.

### 2.3. Study Area Description

**Figure 3:**
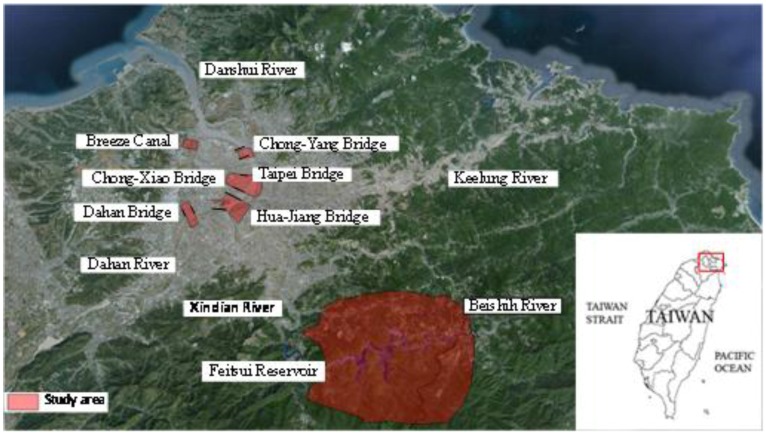
The stream network and basin of Danshui River.

The basin of Danshui River is located in northern Taiwan. The river has a length of 159 km, its basin covers the area of 2,726 km^2^, and the average slope is about 1:122. It is considered the most important stream in Northern Taiwan. Figure 3 shows the network and basin location of Danshui River. There are three main tributaries for Danshui River: Dahan River, Xindian River, and Keeling River. Among these three streams, the north-south oriented Dahan River and Xindian River merge to form the main stream of Danshui River, which is about 21 km in length. Later, after the east-west oriented Keelung River joins the mainstream, Danshui River flows into the Taiwan Strait. Danshui River is a tidal stream. The flow condition is significantly influenced by the tidal variation and thus there are many sandbanks and wetlands formed in the stream. There are two major reservoirs in the stream network: they are the Fetsui reservoir, located at the upstream of Beishi River, and Shimen reservoir, at the upstream of Dahan River. The levees with design return period of 200 years are built in the downstream section of Danshui River, except for the zones close to Taiwan Strait, and in the upstream part of Xindian River, to protect the population of 5 million in the Taipei metropolitan area and also to reduce possible flood damage.

Within the basin, most precipitation falls in the periods between April and May due to plum rain, from July to September because of typhoons, and from December to February of next year due to monsoons. The terrain affects precipitation intensity as the value increases when moving from the plains toward the mountain area, while the windward side has a higher value than the leeward one. A proportional relationship is observed between the flow rate and precipitation distribution. For the wet period between May and October, the flow rate occupies 67 percent of the annual amount in the rest months, considered the dry period, with a share of 33 percent. Most areas that Danshui River flows through have been urbanized. For the Taipei Metropolitan area, the total area is 2,324.37 km^2^; 45.5% of it is categorized as urban use, while Taipei City occupies 271.8 km^2^. The study area is considered a tidal zone and belongs to a developed metropolitan area. The land, used for comparison located near Fetsui Reservoir, is protected by the Taipei Water Management Office and most is covered by trees as a forest.

**Table 1:** Summary of accuracy and error source.

	Observation							
**Estimation**		**Water**	**Bare soil**	**Construction**	**Herbal**	**Plant**	**Total Pixel**	**User Accuracy (%)**
	Water	24	0	0	0	0	24	100
	Bare soil	0	47	5	2	4	58	81
	Construction	1	3	45	0	0	49	92
	Herbal	0	8	7	34	0	49	69
	Plant	0	0	0	0	79	79	100
	total pixel	25	58	57	36	83	259	overall accuracy = 88.4%
	Producer Accuracy (%)	96	81	79	94	95		Kappa = 0.85

## 3. Results and Discussion

### 3.1. Research and Analysis of Surface Temperature

The data was collected by the Landsat-7 and Formosat-2 on 5 June 2005, 26 April 2008, and 12 May 2008. The thermal infrared band was extracted from the Landsat-7 data and later consolidated with the satellite images collected by Formosat-2. This joined information was then used for retrieving the surface temperature.

**Figure 4:**
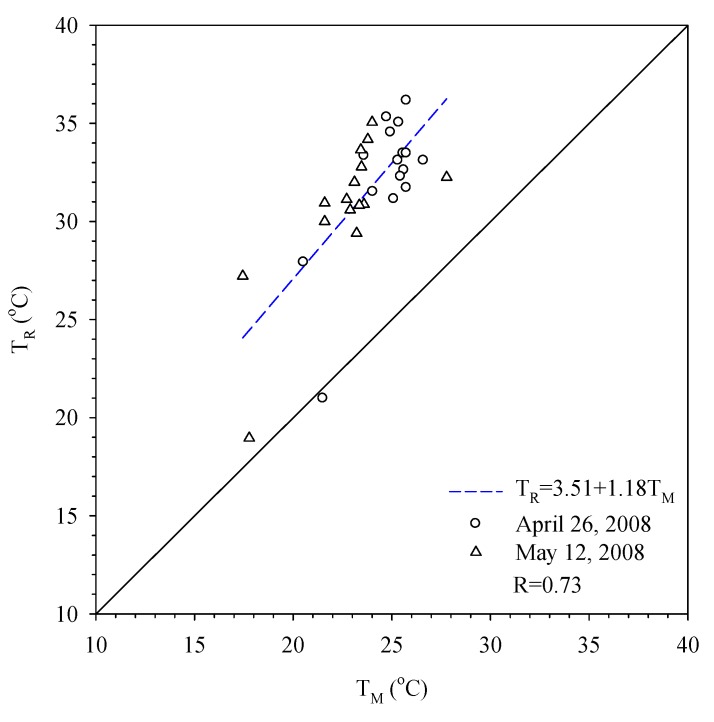
Comparison of temperatures measured by meteorological stations (*T_M_*) and estimated by remote sensing (*T_R_*).

The image resolution of the Landsat-7 is 60 m by 60 m, while for the Formosat-2 it is 8 m by 8 m. This study adopts the 8 m resolution of Formosat-2 data and assigns the long wave radiation rate for each land use; after compiling with the Landsat-7 dataset, the surface temperature at 8 m by 8 m resolution can be derived. Figure 4 shows the surface temperature derived by the satellite data and also its comparison with the air temperature near the surface of the Earth data measured by 17 fixed meteorological stations located in the Taipei Metropolitan area. The derived data *T_R_* shows 10 degrees higher than the numbers collected by the fixed stations *T_M_*. However, the two sets demonstrate a consistent trend; thus, the remote sensing technique can be used reliably to derive the temperature. 

### 3.2. Cooling Effect of Rivers on the Natural Area

**Figure 5:**
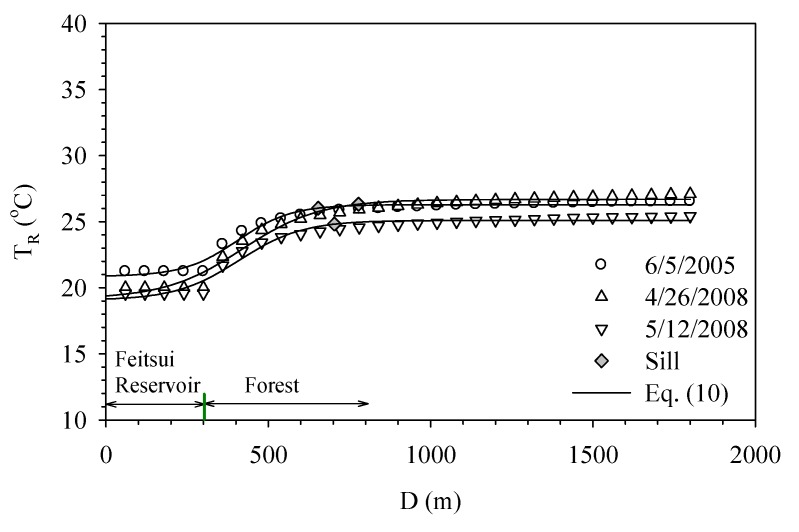
Cooling effect of the Feitsui Reservoir on its ambient natural area.

When the land use contains only a water body and vegetation, the variation in environmental temperature is mitigated by evapotranspiration, a property shared by the two land use categories. In this study, the data within 1,500 meters radius of Fetsui Reservoir near Taipei City is collected for matching with the resolution of the Landsat-7 image data 60 m by 60 m. The purpose is to understand how the water body affects the environmental temperature in a natural setting. A commonly used function for modeling a discontinuity at the origin of the temperature is the sigmoid model:

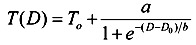
(10)
where *T*(*D*) is the temperature; *D* is the distance from the center of water; *T_0_* is the temperature of the water surface; *a*, *b* and *D_o_* are parameters. The graph of *T*(*D*) against *D* shows the temperature increases with distance. However, the temperature is bounded by a finite value called sill. *T*(*D*) and *T*(*D + h*) are uncorrelated when *D* is larger than sill. The parameter *a* represents the maximum temperature equaling *T_0_* + 0.5*a*, and parameter *b* determines how quickly the temperature rises. Figure 5 shows the temperatures around the Feitsui Reservoir at different distances. The temperature function increases by 95% from the water surface to the maximum temperature when (*D* – *D_0_*) = 3*b*. Thus influence distance is defined the horizontal length between the 95% temperature range. Beyond the influence distance, the surface temperature in urban area is not affected by the cooling effect. When the practical range of the distance from the river bank is between 320 and 388 m, the sigmoid model approaches the sill to 95%. The temperature behind the sill slowly increases. Figure 5 also shows the difference in air temperature between the water and forest is around 5 °C. Therefore, the range of cooling effect by the Beishih River in the natural area near Taipei is around 350 m.

### 3.3. Cooling Effect of River on the Urban Area

The land use in the study area can be categorized as river, city, and the buffer zone in between the wetlands and flood plain. Each sixty meters is considered to be one segment, starting from the river and extending toward the city. The remote sensing technique is used to determine the temperature of each segment and then the possible range of effect for the water body and wetland is determined through Equation (10) and the nonlinear regression method.

**Figure 6:**
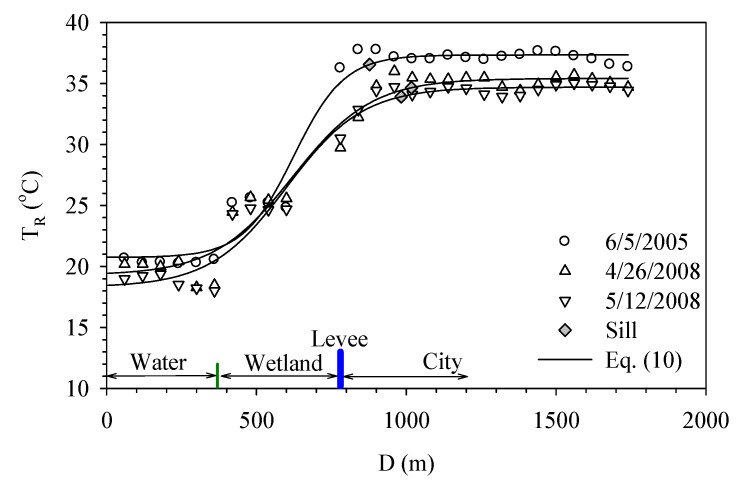
Temperature variation profiles at Hua-Jiang Bridge.

The Hua-Jiang Bridge wetland is located close to the intersection of Dahan River and Xindian River. Danshui River is affected by the ocean current and tide and thus the flow velocity is reduced. The substantial sedimentation results in the formation of a vast wetland. Figure 6 shows the temperature variation around the Hua-Jiang Bridge wetland and also its relation to the distance from Danshui River. It demonstrates the surface temperature shows little change over the water body while a gap of 5 °C exists between the wetland and river. Within the wetland, the variation remains small and the temperature remains around 25 °C. When moving toward the city, the temperature rises gradually to 35 °C. This rise almost comes to a stop as the distance goes beyond 200 meters. 

**Figure 7:**
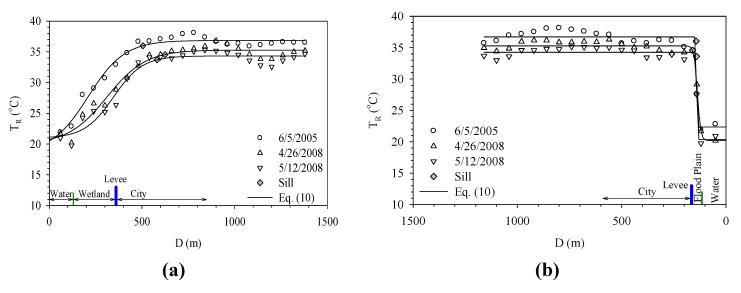
Temperature variation profiles of right bank (**a**) and left bank (**b**) of Xin-Hai Wetland.

Figure 7a shows how Dahan River and Xinhai Constructed Wetland affect the urban temperature. Xinhai Constructed Wetland is next to the right bank of Dahan River and located between Dahan Bridge and Taliouken Creek. The main purpose is for urban wastewater treatment. The constructed wetland is an application of Natural Treatment System principles, taking advantage of the physical, chemical, and biological reactions among the water, soil, plants, microbes, and/or air to improve water quality. The figure shows the temperature rises as the distance from Dahan River increases, while the area with more significant change happens within the wetland. However, beyond 250 meters from the levee, the temperature becomes stable. Figure 7b presents how Dahan River affects the urban temperature of the left bank around Xinhai Wetland. As the width of the flood plain is narrow and the main land use is asphalt paved roadway, the temperature over the flood plain is much higher than the water body and thus an abrupt increase is observed. Moreover, due to the sill being located within the flood plain, it shows the water body does not affect the urban surface temperature in a significant way. Figure 7 overall demonstrates the wetland between the river and city can buffer the temperature variation, while the land use of the flood plain also contributes to how the range of effect varies. 

**Figure 8:**
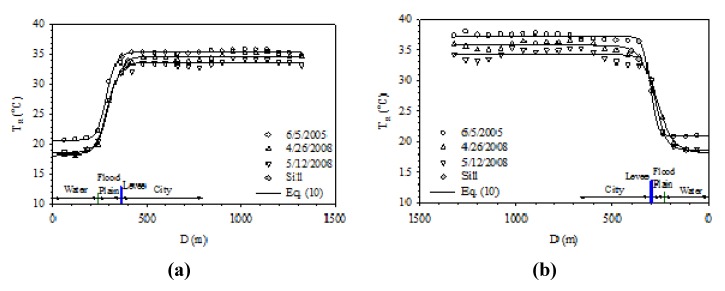
Surface temperature variation profiles of right bank (**a**) and left bank (**b**) at Taipei Bridge and Chong-Xiao Bridge.

Danshui River has the narrowest width around Taipei Bridge. Between the river and the city, there is only a narrow flood plain and levee, while the main land use category over the flood plain is bare land. Beside the paved roadways and parking lots, there is no other development in this zone. Figure 8 shows the effect of Danshui River on the temperature around Taipei Bridge. Figure 8a is the right bank, which belongs to Taipei City. The surface temperature rises rather drastically over the flood plain and then this trend becomes stable after moving beyond the levee. Figure 8b demonstrates how the surface temperature of New Taipei City, at the left bank, is affected. The study area is mostly bare land without any development, a variation similar to what happens in Taipei City; that is, the temperature over the water body and the urban land protected by a levee has little variation, while the main fluctuation occurs within the flood plain.

**Figure 9:**
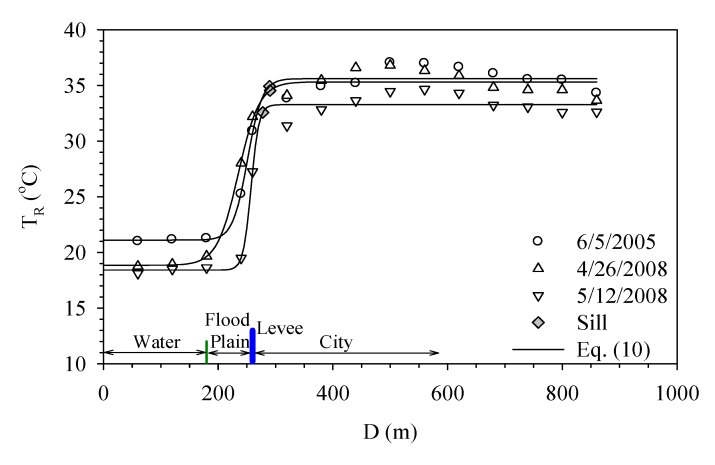
Surface temperature variation at Chong-Yang Bridge Location.

Figure 9 shows how Danshui River affects the urban surface temperature of Taipei City around Chong-Yang Bridge. Most of the flood plain in this area is bare land and paved roads. From Danshui River to the highly developed Taipei City, the temperature rises about 15 °C within a short distance over the flood plain and then stabilizes.

**Figure 10:**
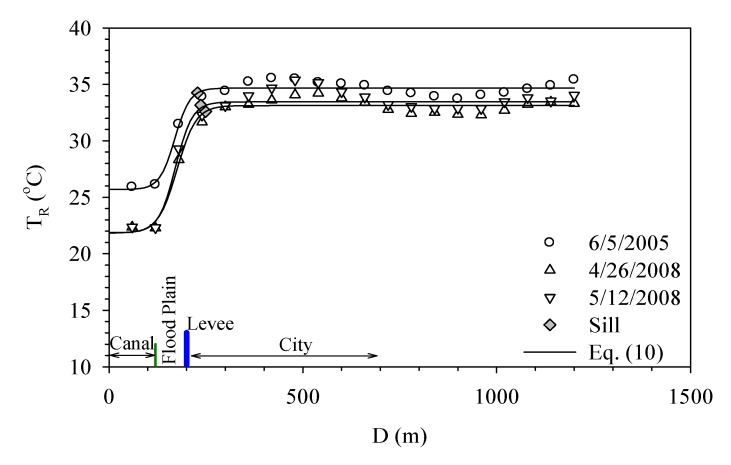
Surface temperature variation at Breeze Canal Location.

**Figure 11:**
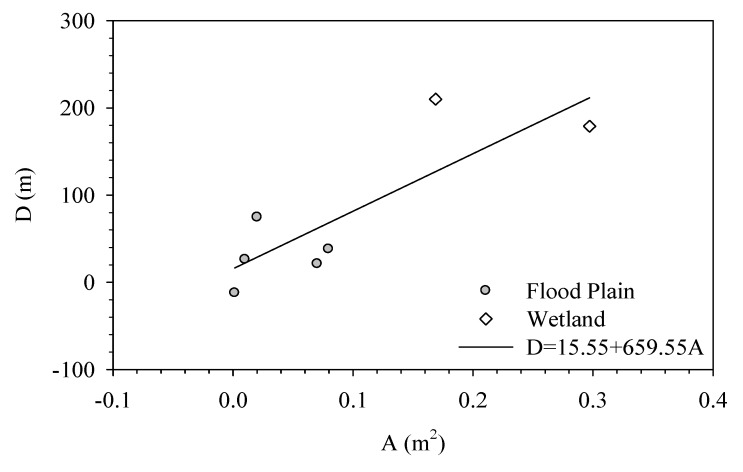
The relationship between the affected range and area of plains and wetlands.

Figure 10 demonstrates how the Breeze Canal affects the temperature at New Taipei City. The Breeze Canal is an artificial canal located at the tail segment of Erchong Floodway. The length is around 1.3 km, while the width is about 123 meters. Its major use is as a training base for windsurfing, canoe, and kayak teams. As shown in the figure, the flood plain and levee separate the river and city, while the main facilities over the flood plain are sports venues, paved roadways, and parking lots. The major difference from other cases is the urban surface temperature drops slightly at a distance between 700 and 1,100 meters. This is caused by an elementary school where the land is developed at a low level while having many trees planted on campus. This indicates variations in land use can result in temperature fluctuations. Table 2 lists how the water body affects the surface temperature. The range of effect is defined as the distance from either the levee or the river bank. For the natural area, the range of effect can reach between 320 and 388 meters. When there are wetlands between the river and levee, the value is about 160 meters. If there are only flood plains in between, the range can vary between −19 and 71 meters, where the negative value means the location before the levee; that is, the surface temperature stabilizes before reaching the levee. The effect of rivers on those areas without wetland is obviously smaller while those areas with wetland have a range of effect between 174 and 217 meters. Figure 11 shows the relationship between the range of effect and either the bare land or wetland, where the x axis is the average range of effect. As observed in the figure, the larger the area of wetland or bare land, the more significant the effect of the river on the surface temperature is. The equation is *D* = 15.55 + 659.55 × *A* while *r^2^* = 0.70. Comparing this result with the effect of natural areas, the effect from rivers is less significant, while such an effect is affected by the size of the wetland or flood plain in the buffer zone.

**Table 2:** Summary of area affected.

Location	Date	Average Channel width (m)	Average Width of Wetland (m)	Average Width of Flood Plain (m)	Influence Distance (m)	Average Influence Distance (m)
Feitsui River	6/5/2005				388	304
	4/26/2008				254	
	5/12/2008				320	
Hua-jiang Estuary	6/5/2005	433	514		97	179
	4/26/2008	462	537		237	
	5/12/2008	437	508		202	
Xin-hai Wetland (Right bank)	6/5/2005	141	237		145	210
	4/26/2008	138	234		264	
	5/12/2008	132	231		221	
Xin-hai Wetland (Left bank)	6/5/2005	138		42	−17	−12
	4/26/2008	132		42	−1	
	5/12/2008	141		43	−19	
Taipei Bridge (Right bank)	6/5/2005	469		102	3	21
	4/26/2008	482		105	37	
	5/12/2008	473		103	23	
Taipei Bridge (Left bank)	6/5/2005	469		30	60	75
	4/26/2008	482		32	102	
	5/12/2008	473		31	62	
Chong-yang Bridge	6/5/2005	74		58	30	26
	4/26/2008	77		56	30	
	5/12/2008	75		57	18	
Wei-fong Canal	6/5/2005	125		89	28	38
	4/26/2008	128		96	49	
	5/12/2008	126		90	37	

## 4. Conclusions

Rivers contribute to the variation in urban surface temperature and can mitigate the negative consequences of the urban heat island. This study utilizes satellite images to evaluate the surface temperature and land use of the Taipei metropolitan area, while analyzing the range of effect from rivers on the surface temperature and its associated factors. Using data from Landsat-7 and Formosat-2, the surface temperature and land use information can be quickly and accurately collected for a large area. Then the range of effect from the river on the surface temperature can be determined. The study finds the river can have a range of effect of around 300 meters for an undeveloped natural area. Moreover, the urban heat island effect causes the difference between the urban and river surface temperatures to be greater than what is observed in the undeveloped zone, while such variation occurs mostly within the buffer zone between the river and city. The high-rises in cities insulate the horizontal thermal dispersion of heat produced within and thus force an upward propagation, resulting in a reduced effect range compared with the natural area and weakening the effect of rivers on urban temperature reduction. Moreover, the land use in the buffer zone also affects the range of effect; the range is smaller when the buffer zone is mainly a flood plain, while such a value is bigger if the buffer zone is occupied by wetland. Nevertheless, the range of effect on the urban surface temperature is related to the area of buffer zone between the city and river; the bigger the buffer zone, the further the range of effect can reach. Though there were several applications of LST retrieved from satellite imagery for the UHI, the authors believed the following innovations still contribute the findings in this field: (1) We integrate different spatial resolutions of two satellite imagery to get more details of land use type to obtain accurate surface information which yields the long-wave radiation reflectivity; (2) from the derived relationships of satellite LST and ground meteorological stations, we have proposed an approach deciding the influence distance of river cooling effect and its relationship with the area of plains and wetlands. Our findings still advance to the knowledge for the Urban Heat Island effect for the future landscape and urban planning.
